# Digoxin exerts anticancer activity on human nonsmall cell lung cancer cells by blocking PI3K/Akt pathway

**DOI:** 10.1042/BSR20211056

**Published:** 2021-10-06

**Authors:** Yingying Wang, Yongqiang Hou, Lanjiao Hou, Wei Wang, Ke Li, Zhe Zhang, Bo Du, Dexin Kong

**Affiliations:** 1 Tianjin Key Laboratory on Technologies Enabling Development of Clinical Therapeutics and Diagnostics, School of Pharmaceutical Sciences, Tianjin Medical University, Tianjin 300070, China; 2 Department of Otorhinolaryngology Head and Neck, Institute of Otorhinolaryngology, Tianjin First Central Hospital, Tianjin 300192, China; 3 Tianjin University Hospital, Tianjin 300072, China; 4 Tianjin Key Laboratory of Biomedical Materials, Biomedical Barriers Research Center, Institute of Biomedical Engineering, Chinese Academy of Medical Sciences & Peking Union Medical College, Tianjin 300192, China; 5 School of Medicine, Tianjin Tianshi College, Tianyuan University, Tianjin 301700, China

**Keywords:** autophagy, digoxin, metastasis, nonsmall cell lung cancer, proliferation

## Abstract

Lung cancer remains the leading cause of cancer mortality because of its metastatic potential and high malignancy. The discovery of new applications for old drugs is a shortcut for cancer therapy. We recently investigated the antitumor effect of digoxin, a well-established drug for treating heart failure, against nonsmall cell lung cancer A549 and H1299 cells. Digoxin inhibited the proliferation and colony-forming ability of the two cell lines and arrested the cell cycle at the G0/G1 phase in A549 cells and the G2/M phase in H1299 cells. Mitochondria-mediated apoptosis was induced in A549 cells but not in H1299 cells after treatment with digoxin. Moreover, digoxin inhibited the migration, invasion, adhesion and epithelial–mesenchymal transition of A549 and H1299 cells. Autophagy was induced in both cell lines after treatment with digoxin, with an increase in autophagosome foci. In addition, digoxin inhibited the phosphorylation of Akt, mTOR and p70S6K, signaling molecules of the PI3K/Akt pathway that are known to be involved in tumor cell survival, proliferation, metastasis and autophagy. Our findings suggest that digoxin has the potential to be used for therapy for human nonsmall cell lung cancer, but further evidence is required.

## Introduction

Lung cancer is the most commonly diagnosed cancer in the world, with approximately 1,762,450 cancer cases diagnosed in 2019 [1]. Nonsmall cell lung cancer (NSCLC) accounts for approximately 85% of lung cancers and has the highest cancer mortality rate [2]. Metastasis is a major cause of morbidity and mortality in NSCLC. Lung carcinomas at the time of diagnosis are most often in the metastatic stage [3,4]. Lung cancer can metastasize to different sites, such as the brain, bone and adrenal glands [5,6]. Unfortunately, current treatments for NSCLC, including surgery, chemotherapy, radiation and targeted therapies, are far from meeting clinical requirements [7]. It is urgent to find more efficient potential antitumor drugs for lung cancer therapy.

Digoxin is widely used to treat patients with congestive heart failure [8–10]. Moreover, its potential antitumor effects have been widely reported [11–14]. Clinical tests of digoxin as an anticancer drug, alone or in combination with other chemotherapeutic drugs, have been reported in recent years [15–17]. Our previous study found that digoxin inhibits the proliferation of nonsmall cell lung cancer cells by inhibiting DNA damage repair. Combination treatment with doxorubicin can significantly increase the antitumor effects and reduce the dose of digoxin [18]. In addition, we found that digoxin inhibits the angiogenesis of human umbilical vein endothelial cells (HUVECs) by inhibiting the secretion of MMP2/9 and reduces the metastasis and invasion of colorectal cancer cells [19]. However, the anticancer mechanisms of digoxin have not yet been fully elucidated.

The phosphoinositide 3-kinase (PI3K)/protein kinase B (AKT)/mammalian target of rapamycin (mTOR) pathway plays a central role in fundamental cellular responses such as cell growth, survival, migration and metabolism. However, key signaling proteins of this pathway are frequently mutated or overexpressed in tumor cells, leading to tumorigenesis as well as metastasis [20–22]. Therefore, inactivation of PI3K and Akt is thought to inhibit the survival, growth and invasion of cancer cells and tumor angiogenesis [21,23]. Digoxin can inhibit the Na^+^/K^+^-ATP enzyme on the cell membrane and inhibit the DNA damage repair pathway in the nucleus, but its effects on PI3K signaling pathway-related intracellular signal transduction are not clear.

In the present study, we investigated the inhibitory effects of digoxin on NSCLC cells and elucidated the molecular mechanisms that account for its therapeutic effect. We demonstrated that digoxin inhibited proliferation and induced cycle arrest and autophagy in A549 and H1299 cells. In addition, digoxin could inhibit the migration and invasion of A549 and H1299 cells by suppressing epithelial–mesenchymal transition (EMT). Moreover, the PI3K/AKT/mTOR signaling pathway was proven to play an important role in the anticancer effect of digoxin on NSCLC. The present study enriches the understanding of the antitumor mechanisms of digoxin and provides a basis for the research and development of this kind of antitumor drug.

## Materials and methods

### Reagents

Digoxin was purchased from Aladdin (London, ON, Canada). Wortmannin was purchased from Selleck (Danvers, MA, U.S.A.). Anti-p27, anti-cyclin D1, anti-Cdc2, anti-cyclin B1, anti-PARP, anti-E-cadherin, anti-ZEB1, anti-vimentin, anti-LC3B, anti-Atg5, anti-p62, anti-Akt, anti-p-Akt (phospho S473), anti-mTOR, anti-p-mTOR (phospho S2448), anti-p-p70S6K (phospho T389) and anti-β-actin antibodies, as well as anti-mouse and anti-rabbit HRP-conjugated secondary antibodies, were obtained from Cell Signaling Technology (Danvers, MA, U.S.A.). The anti-Twist antibody was purchased from Santa Cruz Biotechnology (Santa Cruz, CA, U.S.A.). Matrigel and the annexin V-FITC/PI apoptosis detection kit were from BD Biosciences (San Jose, CA, U.S.A.). Propidium iodide (PI) was from Sigma-Aldrich (St. Louis, MO, U.S.A.).

### Cell culture

Human nonsmall cell lung cancer A549 and H1299 cells were obtained from the Cell Resource Centre, Peking Union Medical College (Beijing, China). The cells were cultured in Roswell Park Memorial Institute (RPMI) 1640 medium supplemented with 10% foetal bovine serum (FBS), 10 μg/ml streptomycin and 100 U/ml penicillin at 37°C in a humidified atmosphere containing 5% CO_2_. All experiments were performed with mycoplasma-free cells.

### Plate colony formation assay

A plate colony formation assay was used to determine the tumorigenicity of A549 and H1299 cells as we reported previously [24]. Cells were seeded into six-well plates at a density of 500 cells per well and cultured with 10% FBS/RPMI 1640 medium supplemented with the indicated concentrations of digoxin. After culture for 8 days at 37°C with 5% CO_2_, the colonies were fixed with 4% paraformaldehyde followed by 30 min of Crystal Violet (0.5%) staining. Colonies larger than 0.1 mm in diameter were counted using ImageJ software (National Institutes of Health, Bethesda, MD, U.S.A.).

### Cell viability assay (peripheral blood mononuclear cells [PBMCs])

The effect of digoxin on cell viability was determined by the MTT assay as outlined in our previous study [25]. In brief, human PBMCs were isolated from 15 ml of peripheral blood through density gradient centrifugation using Lymphoprep (DAKEWE, Shenzhen, China), and the suspensions of PBMCs were seeded into 96-well plates at a density of 8 × 10^3^ cells/200 µl per well. On the following day, different concentrations of digoxin were added to PBMCs. After 24 h of treatment, 20 µl of MTT reagent was added to each well for an additional 4 h of incubation. Then, the supernatant was removed, and 150 µl DMSO was added to dissolve the formazan crystals. The resulting absorbance at 490 nm was measured by using a microplate reader (iMark, Bio-Rad, Hercules, CA, U.S.A.).

### Flow cytometry for cell cycle distribution analysis

Cell cycle distribution was analysed by PI labeling after the cells were treated with digoxin as we previously described [26]. A549 and H1299 cells were seeded into six-well plates (2 × 10^5^ cells/well) and treated with different concentrations of digoxin for 24 h. The cells were fixed in ice-cold ethanol (70%) and finally resuspended in 50 µg/ml PI solution containing 0.5% Triton X-100 and 2% RNase A. After that, the cells were placed in a dark area for 60 min at 4°C and then assessed by a BD Accuri C6 flow cytometer (BD Biosciences, San Jose, CA, U.S.A.).

### Flow cytometry for cell apoptosis analysis

An annexin V-FITC/PI staining assay was employed to evaluate the apoptotic rate as we described previously [27]. A549 and H1299 cells were seeded into six-well plates (2 × 10^5^ cells/well) and treated with different concentrations of digoxin for 24 h. The treated cells were then collected and incubated with annexin V-FITC and PI in the dark for 20 min. Finally, the cells were resuspended in binding buffer and assesed by a BD Accuri C6 flow cytometer (BD Biosciences, San Jose, CA, U.S.A.).

### Transwell migration assay

Transwell chambers (8 μM pore size, Corning, NY, U.S.A.) were used to confirm the in vitro antimigratory effect of digoxin as we previously described [28]. Briefly, 4 × 10^4^ cells in serum-free medium (100 μl) were seeded into the upper chambers, and various concentrations of digoxin were added. The lower chamber was supplemented with 650 μl of RPMI medium containing 10% FBS and the same concentration of digoxin as that in the upper compartment. After incubation at 37°C for 24 h, cells were fixed with 90% ethanol, stained with 0.5% eosin, observed under an Olympus CKX41 microscope and photographed (Olympus, Tokyo, Japan).

### Transwell invasion assay

Transwell invasion assays were used to examine the invasive ability of A549 and H1299 cells as we previously described. The effect of digoxin on the invasive ability of A549 and H1299 cells was investigated by using Transwell chambers, in which the upper chambers were pretreated with Matrigel (BD Biosciences, San Jose, CA, U.S.A.) to form a filmy barrier. The other procedures and data analysis were the same as in the Transwell migration assay.

### Cell adhesion assay

Cell adhesive ability was determined as previously reported [29] with slight modifications. Briefly, 96-well plates were coated with 100 µg/ml fibronectin and incubated overnight at 4°C. The coated wells were washed twice with PBS and incubated for 30 min with 1% BSA. Suspensions of A549 or H1299 cells pretreated with various concentrations of digoxin for 24 h were seeded into the coated wells and incubated for 2 h. The wells were subsequently washed twice with PBS to remove the unattached cells. The adherent cells were then fixed with 90% ethanol and stained with Crystal Violet overnight. Finally, the wells were washed twice with PBS, and 100 µl of 10% acetic acid was added. The absorbance at 570 nm was measured using a microplate reader (Bio Rad, Hercules, CA, U.S.A.).

### Monodansylcadaverine (MDC) staining assay

Monodansylcadaverine (MDC) staining was used to confirm the existence of autophagic vacuoles as reported previously [30]. Briefly, A549 and H1299 cells were separately seeded onto coverslips placed onto a 12-well plate at a density of 1 × 10^5^ cells/ml overnight at 37°C in a 5% CO_2_ atmosphere and then treated with different concentrations of digoxin for 24 h. The treated cells were incubated with fresh medium containing 0.1 mM MDC for 1 h at 37°C. After washing with PBS, the cells were fixed with 4% paraformaldehyde and immediately observed under a fluorescence microscope (BX51, Olympus, Tokyo, Japan). MDC staining was quantified by determining the fluorescence intensity of cells with ImageJ software (National Institutes of Health, Bethesda, MD, U.S.A.).

### Western blot analysis

Western blot analysis was carried out as we described previously [31]. Lysates of cells treated with digoxin or DMSO (control) were prepared. Proteins in the cell lysates were separated by sodium dodecyl sulfate-polyacrylamide gel electrophoresis (SDS-PAGE) and then transferred onto polyvinylidene fluoride membranes (Millipore, Billerica, MA, U.S.A.). After being blocked, the membranes were incubated with each primary antibody and then with the respective secondary antibody. The blots were visualized with enhanced chemiluminescence (ECL) system and analyzed using Image J software (National Institutes of Health, Bethesda, MD, U.S.A.). The signals were quantified by comparison to that of β-actin, which is widely used as the internal reference in Western blot.

### Statistical analysis

Data are presented as the mean ± SD, representative of at least three independent experiments. One-way ANOVA was used to determine the statistical significance of differences between groups. All statistical analyses were performed using SPSS Statistics (IBM, Armonk, NY, U.S.A.), and differences were considered statistically significant when the *P*-value was less than 0.05.

## Results

### Digoxin reduced the viability of nonsmall cell lung cancer cells *in vitro*

**Figure 1:**
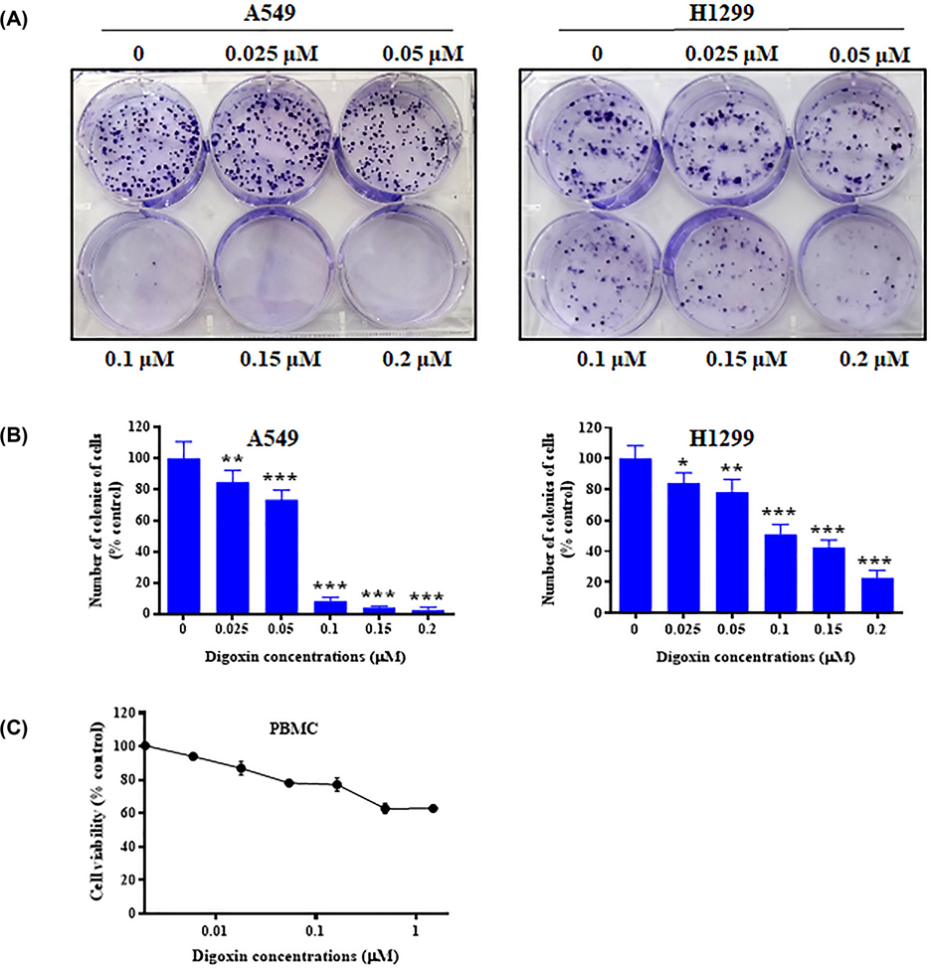
The growth inhibitory effect of digoxin on nonsmall cell lung cancer cells and peripheral blood mononuclear cells (**A**) A plate colony formation assay was used to assess the effect of digoxin on the colony formation capability of A549 and H1299 cells. The cells were incubated for 12 days and then stained with Crystal Violet after treatment with various concentrations of digoxin. (**B**) The histograms represent the number of colonies of A549 and H1299 cells following treatment with digoxin compared with those of control cells. (**C**) PBMCs were assessed by an MTT assay. The data are expressed as mean ± SD (*n*=3); ∗*P<*0.05, ∗∗*P<*0.01, ∗∗∗*P<*0.01, compared with the control.

To investigate the toxic effect of digoxin on nonsmall cell lung cancer cells, we assessed the cellular viabilities of A549 and H1299 cells treated with digoxin at various concentrations by an MTT assay and a plate colony formation assay. Previously, we reported the IC50 values of digoxin on A549 (0.10 μM) and H1299 (0.12 μM) cells in the MTT assay [18]. In the present study, a plate colony formation assay was conducted to further evaluate the tumor growth inhibitory effect of digoxin. As shown in Figure 1A,B, colony formation was significantly inhibited after digoxin treatment of both A549 and H1299 cells in a dose-dependent manner compared with the control group, indicating that digoxin could inhibit the colony-forming abilities of A549 and H1299 cells. Furthermore, digoxin showed a far lower cytotoxic effect on PBMCs, as shown in Figure 1C.

### Digoxin induced cell cycle arrest in nonsmall cell lung cancer A549 and H1299 cells

**Figure 2:**
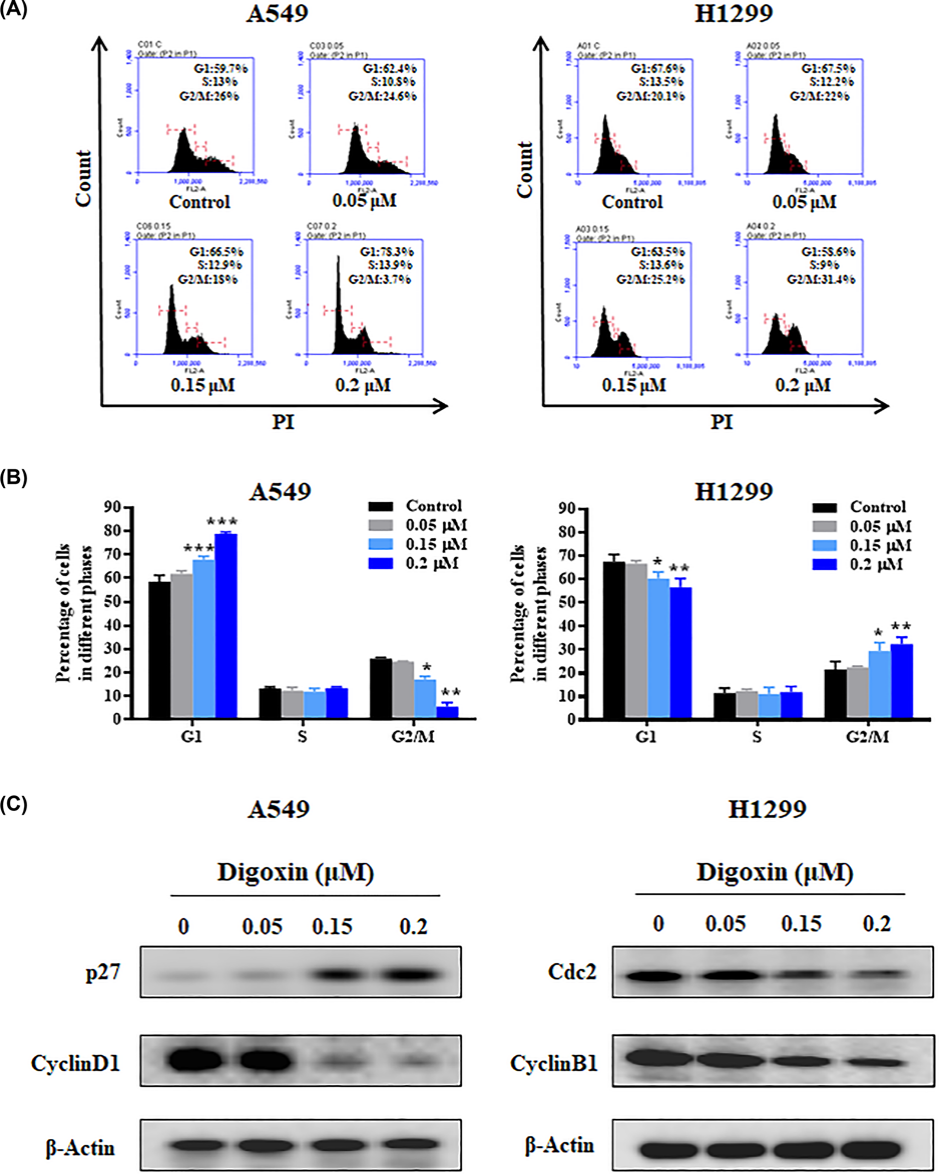
Digoxin induced G1 and G2/M phase arrest in A549 and H1299 cells, respectively (**A**) A549 and H1299 cells were incubated with various concentrations of digoxin for 24 h, then the cell cycle distribution was determined by flow cytometry. (**B**) The percentages of the cell population in G1, S, and G2/M phases. (**C**) Western blotting analysis of the cell cycle-related proteins in cellular extracts from A549 and H1299 cells treated with various concentrations of digoxin for 24 h. The data are expressed as mean ± SD (*n*=3); ∗*P*<0.05, ∗∗*P*<0.01, ∗∗∗*P*<0.01, compared with control.

To study the antiproliferative mechanism of digoxin in nonsmall cell lung cancer cells, we tested whether digoxin treatment affected the nonsmall cell lung cancer cell cycle. A549 and H1299 cells were cultured with different concentrations of digoxin for 24 h, and the cell cycle distribution was detected by flow cytometry. As shown in Figure 2A,B, the cell populations in the G1 and G2/M phases were significantly higher than those of the control group for the A549 and H1299 cells, respectively. To determine how digoxin induces cell cycle arrest in nonsmall cell lung cancer cells, we examined the expression of cell cycle arrest-related proteins through Western blotting. Figure 2C and Supplementary Figure S5a shows that p27 expression was up-regulated while cyclin D1 levels were significantly down-regulated in A549 cells following digoxin treatment. In addition, H1299 cells treated with digoxin showed a significantly reduced expression of Cdc2 and cyclin B1 in a dose-dependent manner.

### Digoxin induced apoptosis of A549 cells

**Figure 3:**
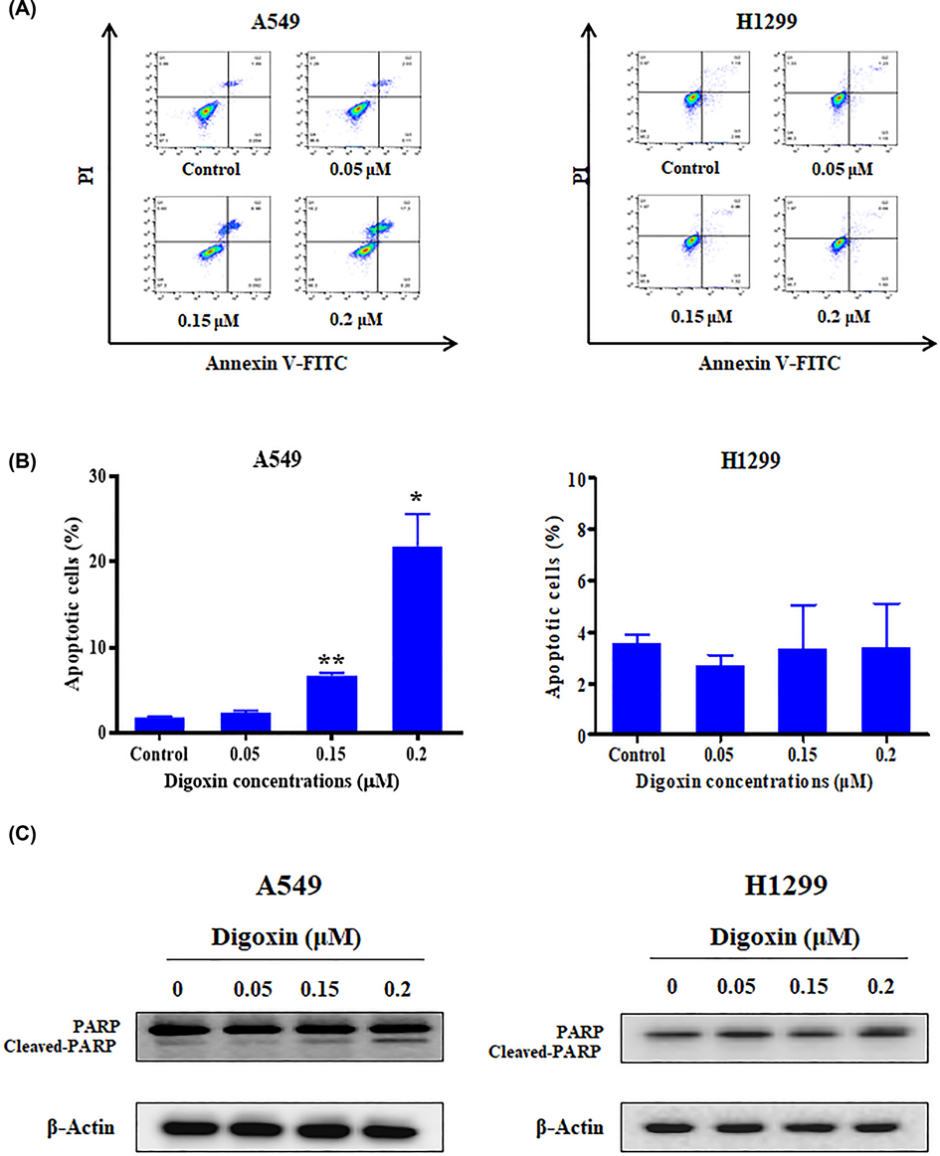
Digoxin induced apoptosis of A549 cells (**A**) A549 and H1299 cells were treated with various concentrations of digoxin for 24 h, stained with Annexin V-FITC and PI, and then measured by flow cytometer. (**B**) Quantification of the apoptotic cells. (**C**) Western blotting analysis of the indicated proteins in cellular extracts from A549 and H1299 cells treated with various concentrations of digoxin for 24 h. The data are expressed as mean ± SD (*n*=3). ∗*P<*0.05, ∗∗∗*P<*0.01, compared with control.

To assess the proapoptotic effect of digoxin, A549 and H1299 cells were treated with different doses of digoxin for 24 h. Apoptotic cells were measured by an annexin V/PI staining assay. As shown in Figure 3A,B, digoxin induced apoptosis of A549 cells. In contrast, there was no significant difference in the number of H1299 cells in the upper right and lower right quadrants compared with the control group after treatment with digoxin. In addition, mitochondria play a key role in keeping cells alive, and interruption of mitochondrial function triggers apoptosis [24]. Therefore, we explored the changes in MMP after digoxin treatment using the membrane permeable fluorescent probe JC-1. As shown in Supplementary Figure S1, digoxin decreased the mitochondrial membrane potential of A549 cells but had no obvious effect on H1299 cells. To verify these results, we examined the changes in apoptosis-related proteins in A549 and H1299 cells induced by digoxin through Western blotting. Figure 3C and Supplementary Figure S5b shows that digoxin treatment increased the cleavage of PARP in A549 cells. However, there was no significant change in the cleavage level of PARP in H1299 cells compared with the control group. The above results showed that digoxin could induce apoptosis in A549 cells but not in H1299 cells.

### Digoxin exhibited antimetastatic activity on NSCLC A549 and H1299 cells that was related to the inhibition of EMT

**Figure 4:**
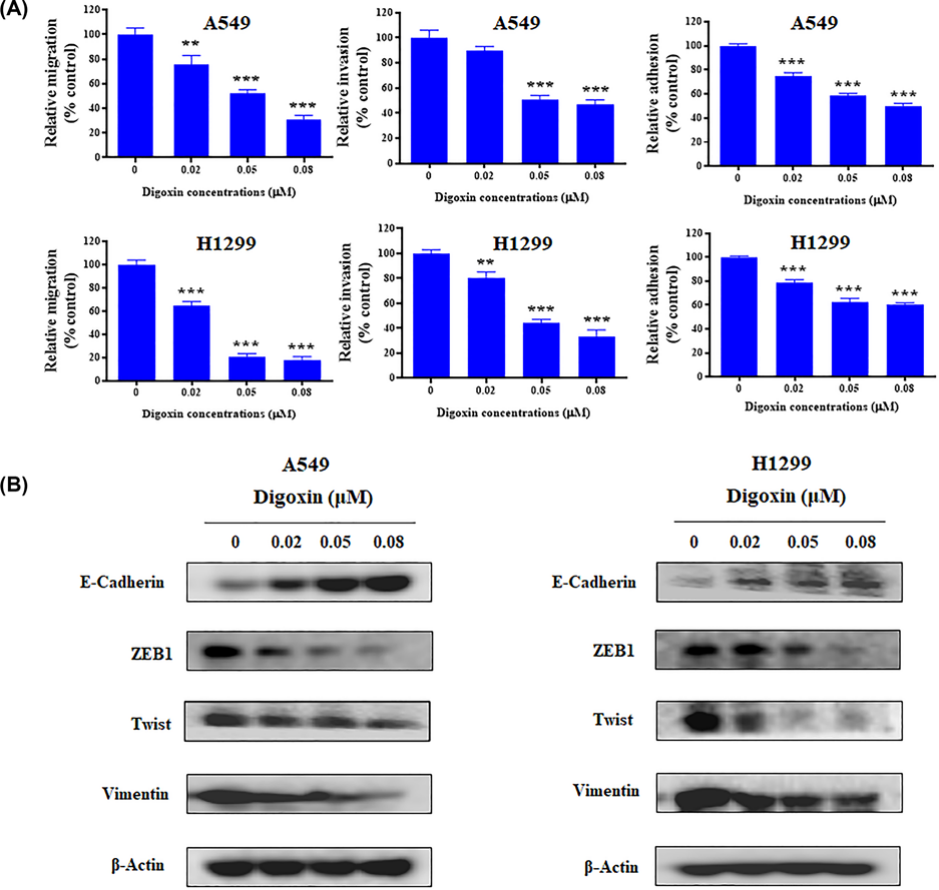
Digoxin inhibited migration, invasion and adhesion of A549 and H1299 cells (**A**) Percentages of A549 and H1299 cells migrated, invaded or adhesion following digoxin treatment relative to those of the control cells. The data are expressed as mean ± SD (*n*=3). ∗*P*<0.05, ∗∗*P*<0.01, ∗∗∗*P*<0.01, compared with control. (**B**) The level of E-cadherin, ZEB1, Twist and Vimentin were detected by used Western blot assay.

To investigate the *in vitro* antimetastatic activity of digoxin, we examined the effect on the migration, invasion and adhesion of A549 and H1299 cells using Transwell migration, invasion and cell adhesion assays, respectively. As shown in Figure 4A, in the Transwell migration assay, digoxin inhibited the migration of A549 and H1299 cells after treatment for 24 h. In addition, the wound healing experiment exhibited a similar result (Supplementary Figure S2), indicating that digoxin inhibited the migration of A549 and H1299 cells in a dose-dependent manner. Then, we used a Transwell invasion assay to detect the effect of digoxin on the invasive ability of A549 and H1299 cells. After treatment with 0.02, 0.05 and 0.08 μM digoxin, the number of A549 and H1299 cells invading through the membrane was reduced remarkably, indicating that digoxin could block the invasion of A549 and H1299 cells in a dose-dependent manner (Figure 4A). Finally, we evaluated the effect of digoxin on the adhesive ability of A549 and H1299 cells. Digoxin treatment for 24 h reduced the number of cells adherent to fibronectin-coated wells in a dose-dependent manner (Figure 4A). To further investigate whether digoxin inhibited NSCLC cell motility through EMT, we evaluated the expression of related epithelial and mesenchymal markers. Western blot analyses demonstrated that digoxin upregulated mesenchymal marker expression levels of E-cadherin while down-regulating protein expression of the mesenchymal-like markers ZEB1, vimentin and Twist (Figure 4B and Supplementary Figure S5c).

### Digoxin induced autophagy in NSCLC cells

**Figure 5:**
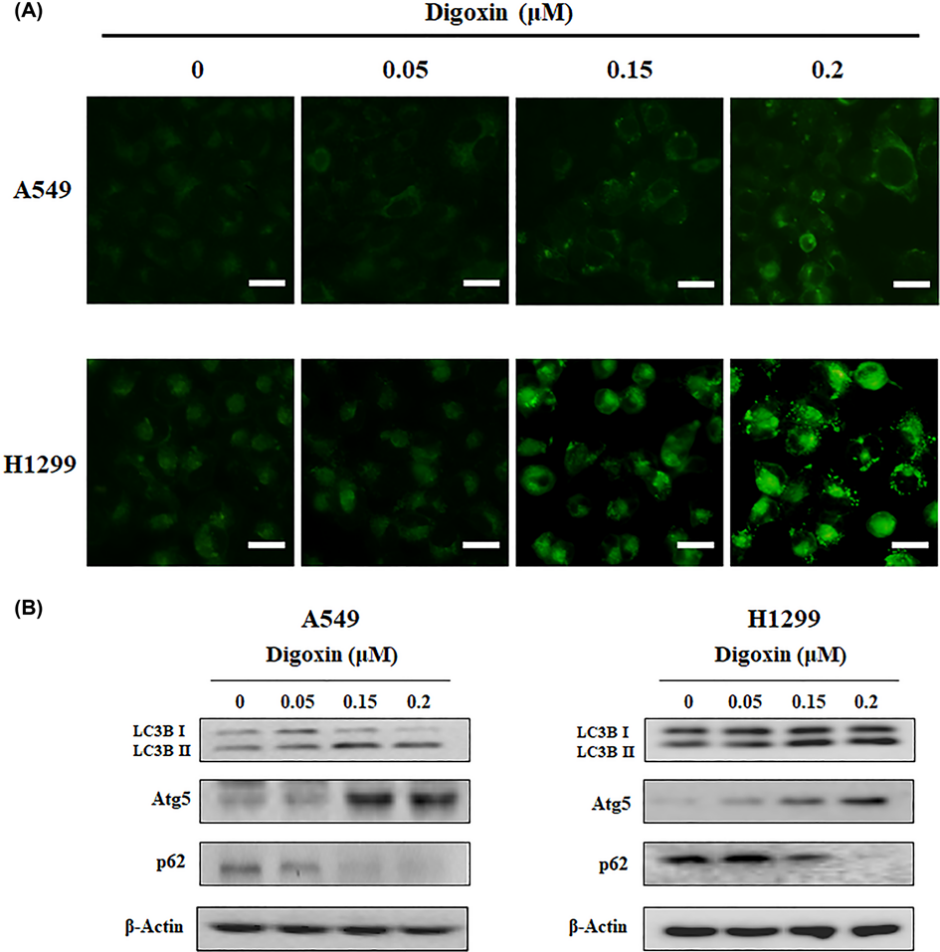
Digoxin induced autophagy of A549 and H1299 cells (**A**) A549 and H1299 cells were treated with indicated concentrations of digoxin for 24 h and then stained with MDC. (**B**) The levels of LC3B, Atg5 and p62 were detected by western blot assay.

To investigate whether digoxin could induce autophagy in A549 and H1299 cells, we performed MDC staining and assessed the expression of LC3-II, Atg5 and p62 in NSCLC cells by Western blotting. MDC is an autofluorescent substance that selectively accumulates in acidic vesicular organelles (AVOs) and is therefore used as a marker for autophagy. After staining with MDC, A549 and H1299 cells treated with or without digoxin were observed under a fluorescence microscope. As shown in Figure 5A and Supplementary Figure S6, the number of autophagic vacuoles increased in these cell lines dose-dependently after treatment with digoxin, suggesting that digoxin induced autophagy in NSCLC cells. Furthermore, the expression of autophagy marker proteins, including LC3B II, p62 and Atg5, was determined by Western blot analysis. As shown in Figure 5B and Supplementary Figure S5d, after treatment with digoxin for 24 h, the conversion of LC3B I to II and the expression of Atg5 increased, while the expression of p62 decreased, further demonstrating the autophagy-inducing activity of digoxin.

### Digoxin blocked the PI3K/Akt/mTOR pathway in NSCLC cells

**Figure 6:**
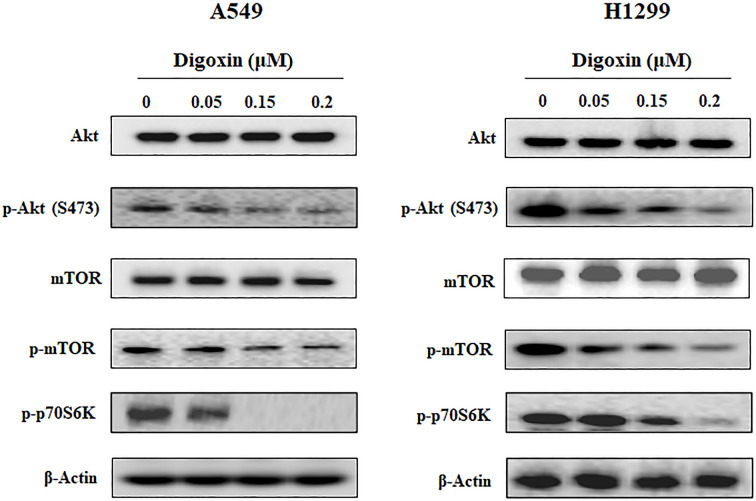
Digoxin blocked the PI3K/Akt/mTOR pathway in A549 and H1299 cells A549 and H1299 cells were treated with indicated concentrations of digoxin for 24 h, then the cells were harvested, and the cell lysates were prepared to be available for Western blot analysis for p-Akt, Akt, mTOR, p-mTOR and p-p70S6K.

The PI3K/Akt/mTOR pathway plays important roles in regulating the cell cycle, cell apoptosis, autophagy and metastasis. To investigate whether the anticancer activity of digoxin was due to class I PI3K inhibition, the effect of digoxin on the representative signaling proteins in that pathway was analyzed by Western blotting. As shown in Figure 6 and Supplementary Figure S5e, the phosphorylation of Akt, mTOR and p70S6K was inhibited dose-dependently after digoxin treatment for 24 h.

## Discussion

Digoxin is a traditional clinical medicine for the treatment of heart failure [32–34]. In addition, digoxin and other cardiac glycosides have been assessed in clinical trials against different types of tumors [17,35,36]. Clinical studies have reported that the recurrence rate in breast carcinoma patients who received cardiac glycosides was lower than in those who did not [37].

Digoxin has been reported to be an inhibitor of the Na^+^/K^+^-ATP enzyme on the cell membrane. Our recent work found that digoxin could inhibit the DNA damage repair pathway in the nucleus of cancer cells [18]. Furthermore, digoxin significantly reversed ABCB1-mediated multidrug resistance in SW620/Ad300 cells [19]. In the present study, we found that digoxin exerted antitumor activities on NSCLC A549 and H1299 cells by inhibiting cancer cell proliferation and inducing cell arrest and autophagy. However, digoxin induced apoptosis in A549 cells but not in H1299 cells and induced cell arrest in different phases of A549 and H1299 cells. These differences may be due to the mutation of TP53 [38]. TP53 encodes p53, and p53 is a transcription factor that regulates the cell cycle and apoptosis [39,40]. Cardiac glycosides have been demonstrated to induce H460 (p53-positive) arrest at the G0/G1 phase and apoptosis, which was similar to the effect of digoxin on A549 (p53-positive) cells [41].

Moreover, digoxin suppressed the metastasis of NSCLC cells by blocking EMT. EMT is a biological process that is essential to cancer invasiveness and metastasis. During this process, cancer cells lose their epithelial characteristics and gain mesenchymal-like features, thus increasing cellular migration and invasion [42]. Our experimental data demonstrated that digoxin inhibited NSCLC cellular motility through EMT reprogramming.

Autophagy is a highly conserved catabolic process that plays important roles in many physiological and disease processes. Cardiac glycosides have been reported to induce autophagy in A549 and H460 cells through regulation of both the mTOR and ERK1/2 signaling pathways [43,44]. Here, we found that digoxin also induced autophagy in A549 cells.

Finally, we investigated the molecular mechanism associated with the above effects of digoxin. Previous reports have shown that digoxin sensitizes gemcitabine-resistant pancreatic cancer cells to gemcitabine by inhibiting Nrf2 signaling through suppressing the PI3K/Akt signaling pathway in SW1990/Gem and Panc-1/Gem cells [45,46], which led us to assess the effect of digoxin on the PI3K/Akt pathways in A549 and H1299 cells. Our results showed that the phosphorylation of PI3K downstream effectors, including Akt, mTOR and p70S6K, was inhibited by digoxin in a dose-dependent manner, while the expression of Akt and mTOR was not changed. Since the PI3K/Akt pathway is aberrantly activated in many cancers and plays a central role in tumor cell proliferation, the antiproliferative effect of digoxin in NSCLC cells might be attributed to its inhibition of the PI3K/Akt pathway. For cancer metastasis, it is well known that the PI3K/Akt pathway regulates EMT master regulators such as ZEB1/2, Snail, Slug and Twist. Inhibition of PI3K by chemical (wortmannin) or genetic (siRNA of PI3K) approaches reduced proliferation and migration of A549 and H1299 cells (Supplementary Figures S3 and S4). Therefore, the antimetastatic effect of digoxin in NSCLC cells might also be attributed to the inhibition of the PI3K/Akt pathway. The PI3K/AKT/mTOR pathway, as a critical regulator of autophagy, is involved in the initiation and promotion of a series of pathological disorders, including various tumors. In the present study, we demonstrated that digoxin induced autophagy in NSCLC cells and inhibited the phosphorylation of AKT and mTOR. It is possible that digoxin-induced autophagy in NSCLC cells is mediated by down-regulation of the PI3K/AKT/mTOR pathway.

## Conclusions

Our results demonstrated that digoxin could significantly inhibit proliferation and metastasis and induce apoptosis and autophagy in NSCLC cells. The mechanism might be attributed to the suppression of the PI3K/Akt signaling pathway. Since digoxin showed weak cytotoxicity on normal cells, it is a promising candidate for NSCLC cancer therapy.

## Supplementary Material

Supplementary Figures S1-S7

## Data Availability

All data generated or analyzed during this study are included in this published article.

## Abbreviations

AKT: protein kinase B
ECL: enhanced chemiluminescence
HUVEC: human umbilical vein endothelial cell
MDC: monodansylcadaverine
mTOR: mammalian target of rapamycin
NSCLC: nonsmall cell lung cancer
PI3K: phosphoinositide 3-kinase
