# Supplementary material for: Digoxin exerts anticancer activity on human nonsmall cell lung cancer cells by blocking PI3K/Akt pathway
Source: Biosci Rep. 2021 Oct 6;41(10):BSR20211056. doi: 10.1042/BSR20211056 (PMC8495431; doi:10.1042/BSR20211056)
Supplement: Supplementary Figures S1-S7 [file BSR-2021-1056_supp.pdf]

Digoxin Exerts Anticancer Activity on Human Nonsmall Cell Lung Cancer Cells by Blocking PI3K/Akt Pathway

Yingying Wang[1‡], Yongqiang Hou[1‡], Lanjiao Hou[1], Wei Wang[2], Ke Li[3], Zhe Zhang[1]*, Bo Du[4]*, Dexin Kong[1,5]*

[1] Tianjin Key Laboratory on Technologies Enabling Development of Clinical Therapeutics and Diagnostics, School of Pharmaceutical Sciences, Tianjin Medical University, Tianjin 300070, China

[2] Department of Otorhinolaryngology Head and Neck, Institute of Otorhinolaryngology, Tianjin First Central Hospital, Tianjin 300192, China

[3] Tianjin University Hospital, Tianjin 300072, China

[4] Tianjin Key Laboratory of Biomedical Materials, Biomedical Barriers Research Center, Institute of Biomedical Engineering, Chinese Academy of Medical Sciences & Peking Union Medical College, Tianjin 300192, China

[5] School of Medicine, Tianjin Tianshi College, Tianyuan University, Tianjin 301700, China

* Corresponding Author E-mail: zhangzhe@tmu.edu.cn; dubo@bme.pumc.edu.cn; kongdexin@tmu.edu.cn.

Materials and Methods

Measurement of mitochondrial membrane potential (MMP)

The changes of MMP were estimated by using [5,5,6,6-Tetrachloro-1,1,3,3-tetraethylbenzimidazolylcarbocyanine iodide] (JC-1) probe as we previously described (Chen et al., 2017) with a little modification. A549 and H1299 cells were seeded into 6-well plates at a density of 2×10^5 cells/mL per well and incubated with different concentrations of digoxin for 24 h. Then the cells were collected and washed by PBS, followed by another 20 min exposure to 2 μM of JC-1 at 37°C in darkness. The samples were suspended with 500 μL of JC-1 staining buffer and the changes of MMP were analyzed by flow cytometer BD Accuri C6 (BD Biosciences, San Jose, CA, USA).

Wound healing assay

Wound healing assay was used to assess the effect of digoxin on cell migration as we previously described (Zhao et al., 2013). A549 and H1299 cells were seeded into 12-well plates (2×10^5 cells per well) and cultured until confluent. The monolayer cells were scratched with a sterile 10 μL pipette tip followed by washing with PBS to remove the floating cells. These cells were treated different concentrations with digoxin for 24 h. The migrated cells were monitored and imaged under the Olympus CKX41 microscope (Olympus, Tokyo, Japan). The migration rate was calculated according to the following equation: migration rate (%) = [test counts/control counts (treated with DMSO)] × 100%.

Small interfering RNA transfection

One hundred picomoles of predesigned small interfering RNA (siRNA) against PIK3CD (Thermo Fisher Scientific) was transfected into NSCLCs cells (2×10^5/well) in 6-well plates using Lipofectamine 2000 in serum-free Opti-MEM according to the manufacturer’s instructions (Thermo Fisher Scientific). The siRNA sequences used were as follows:

PIK3CDsiRNA: sense 5'-CAGAUGAGAAGGGCGAGCUGCUGAA-3' and
anti-sense 5'-UUCAGCAGCUCGCCCUUCUCAUCUG-3'.

Transfected cells were suspended in regular medium containing 10% fetal bovine serum and incubated for further 24 h. The scrambled siRNA was used as a negative control. To detect silencing of p110δ, the whole-cell lysates were subjected to Western blot analysis with anti-p110δ antibody.

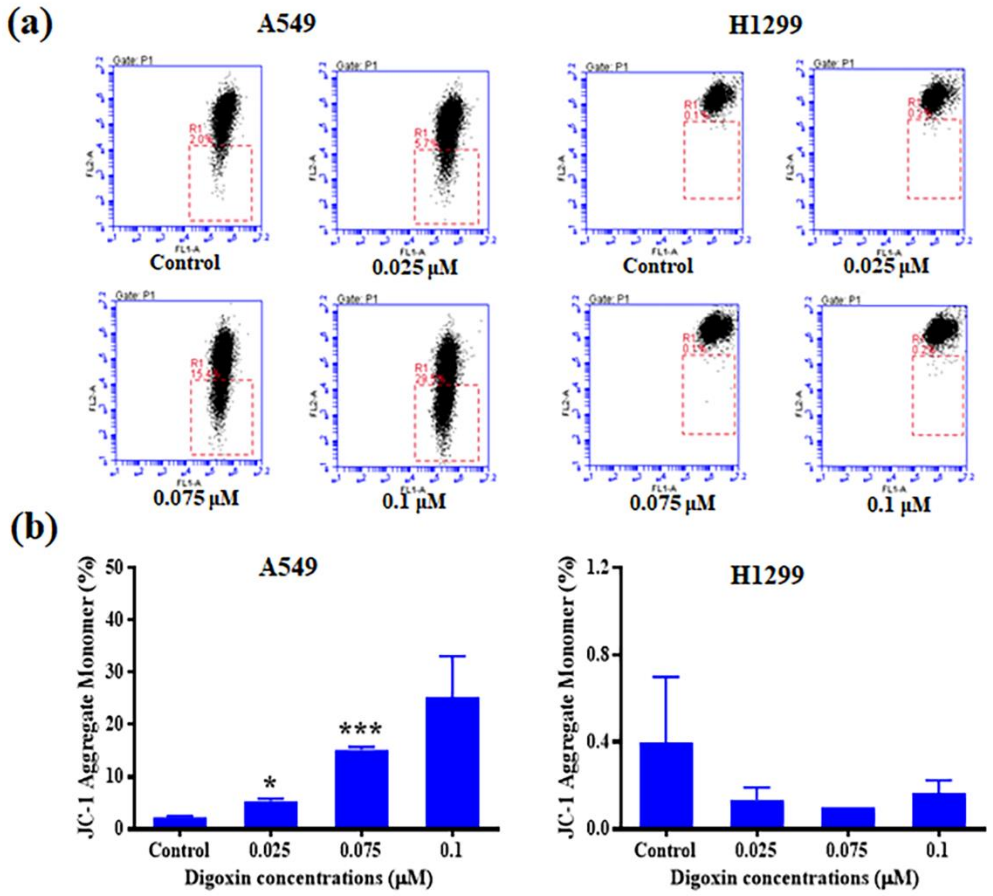

Fig.S1 **Digoxin reduced mitochondrial membrane potential.** (a) Cells were treated with different concentrations of digoxin for 24 h. The MMP was evaluated by staining with the potential sensor JC-1 and analyzed with flow cytometry. Representative dot plot (JC-1 aggregated red fluorescence and monomer green fluorescence) out of four is shown. (b) Dose-dependent changes in the mean JC-1 fluorescence after A549 and H1299 cells were treated with digoxin for 24 h. The results represent mean ± SD of three independent experiments, $*P < 0.05$, $***P < 0.001$, compared with control.

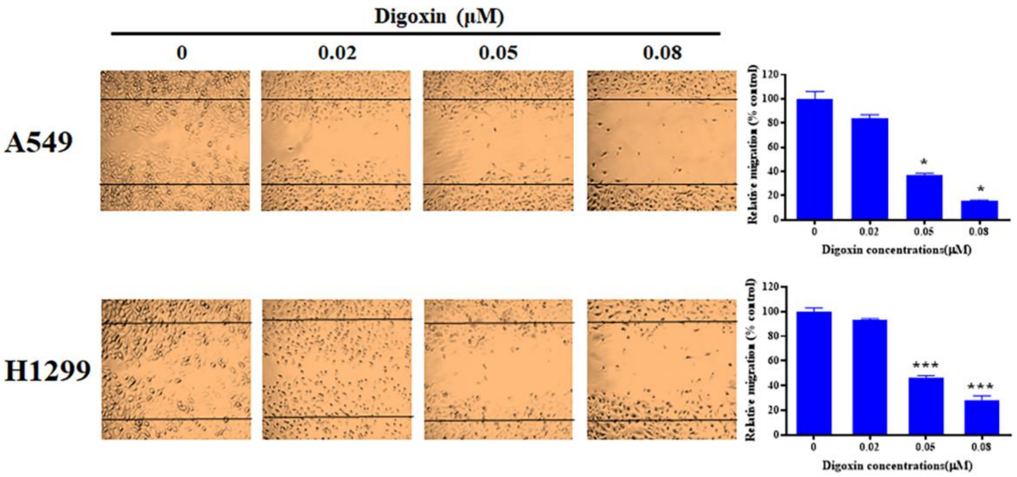

Fig.S2 **Digoxin inhibited migration of A549 and H1299 cells.** Representative images depicting the healing ability of A549 and H1299 cells with or without digoxin treatment after wounded by a 0.2 mL pipette tip, Percentages of A549 and H1299 cells migrated to the wound area following digoxin treatment relative to those of the control cells. Data are mean ± SD (n = 3). $*P < 0.05$, $***P < 0.01$, compared with control.

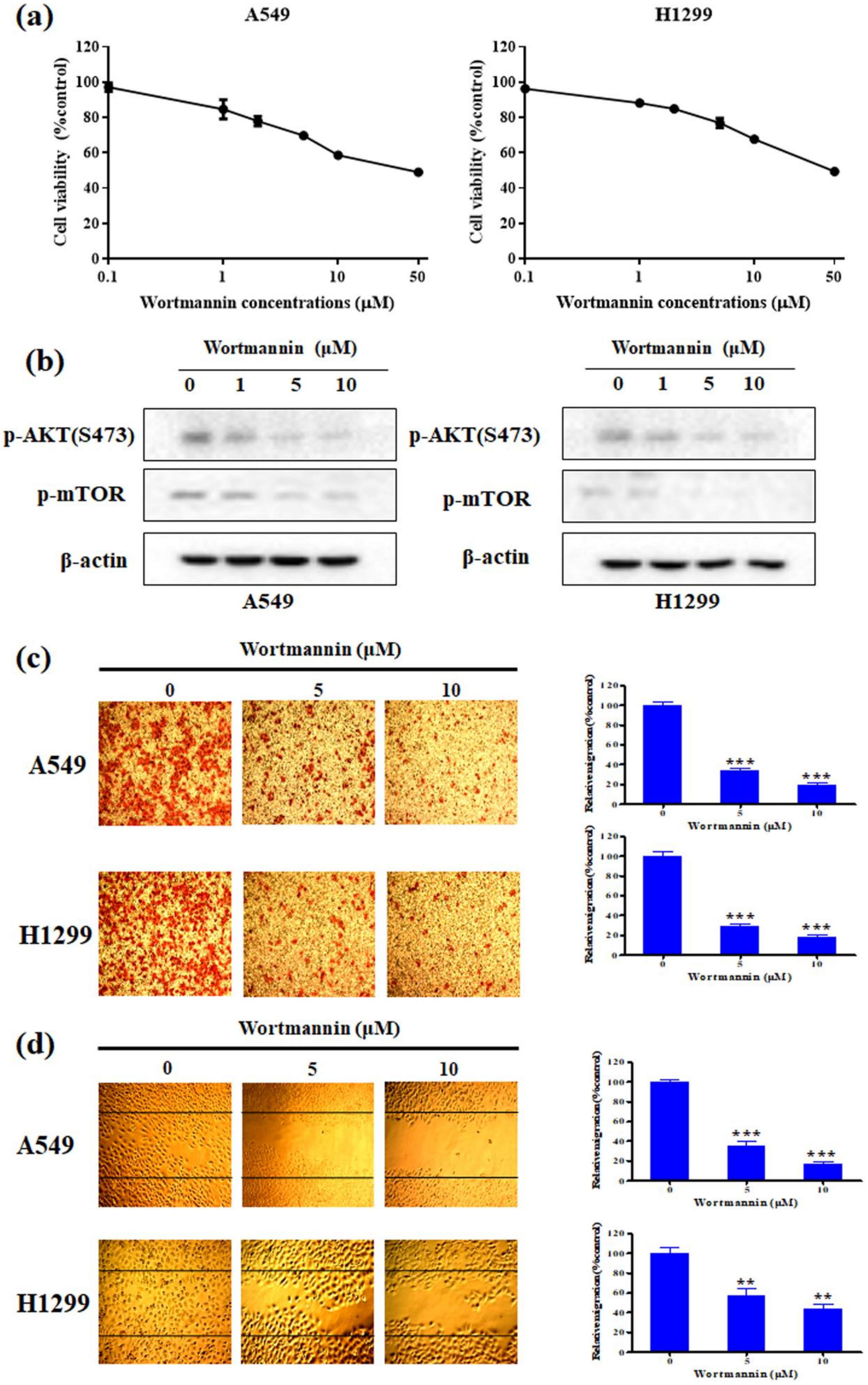
(a)
A549
H1299
Cell viability (%control)
Wortmannin concentrations (μM)
0.1
1
10
50
(b)
Wortmannin (μM)
0 1 5 10
p-AKT(S473)
p-mTOR
β-actin
A549
H1299
(c)
Wortmannin (μM)
0 5 10
A549
H1299
(d)
Wortmannin (μM)
0 5 10
A549
H1299
***
**

Fig.S3 **The inhibition of proliferation and metastasis in NSCLC cells by PI3K inhibitor wortmannin.** (a) A549 and H1299 cells were treated with various concentrations of wortmannin. The cell viability was determined using MTT assay. (b) Wortmannin inhibited the phosphorylation of Akt, mTOR signaling molecules of the PI3K/Akt pathway on both A549 and H1299 cells, the statistical analysis data are shown in Fig.S5f. (c) Cell migration potency was assessed by transwell migration assay. After treatment with wortmannin or DMSO, the cells migrated through the transwell chamber membrane were counted. The relative migration (% control) was calculated by comparison with the control (DMSO) group. (d) Migration of cells was determined using wound healing assay. Cells migrated to the wound area were photographed and counted by inverted microcopy. The relative migration (% control) was calculated by comparison with the control (DMSO) group. The data are expressed as mean ± SD ($n = 3$). $**P < 0.01$, $***P < 0.01$, compared with the control.

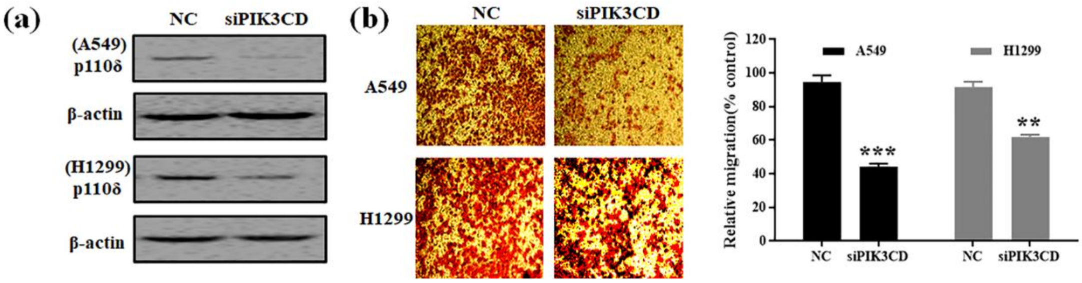

Fig.S4 **The effect of knocking down *PIK3CD* on migration of A549 and H1299 cells.** (a) The changes in expression of PI3K-p110δ in A549 and H1299 cells with PIK3CD knocked down by siRNA. (b) Cell migration potency was assessed by transwell migration assay. The relative migration represents the migratory ability of A549 and H1299 cells with p110δ siRNA treatment, compared with the control cells. The data are expressed as mean ± SD (n = 3), **: p< 0.01, ***: p< 0.001, compared with control.

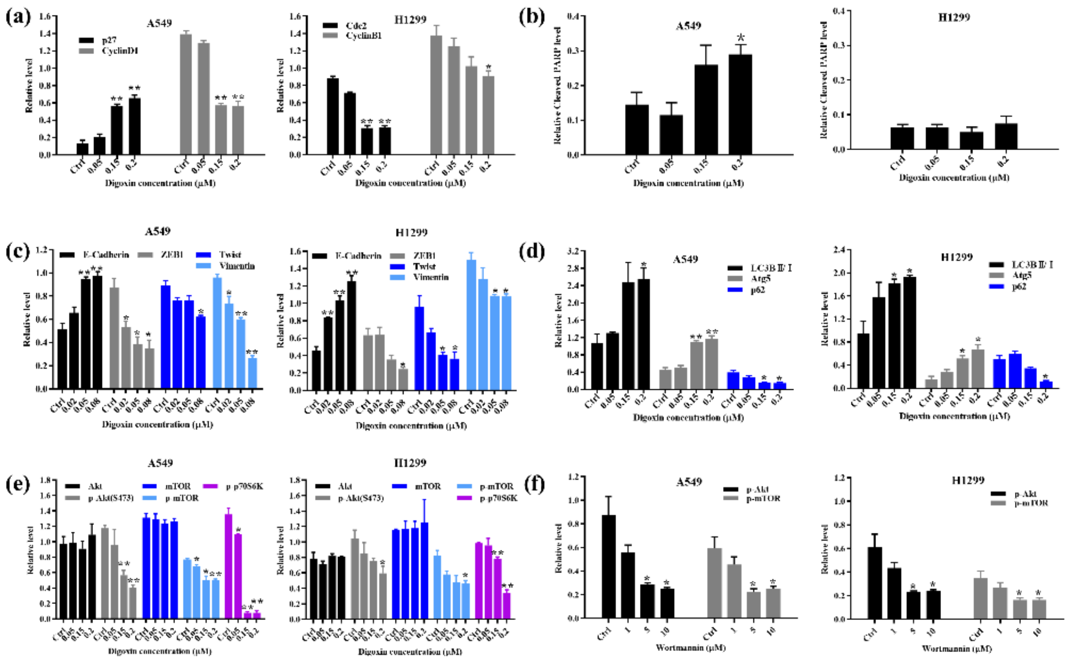

Fig.S5**. The Statistical analysis data of Western blot**. Bar graphs show the relative levels of p27 and CyclinD1 in A549 cells, as well as Cdc2 and CyclinB1 in H1299 cells (a); the relative levels of Cleaved-PARP (b); E-Cadherin, ZEB1, Twist and Vimentin (c); LC3BII/I, Atg5, and p62 (d); Akt, p-Akt, mTOR, p-mTOR and p-p70S6K in A549 and H1299 cells (e), after treatment with digoxin; the relative levels of p-Akt and p-mTOR in A549 and H1299 cells after treatment with Wortmannin (f). The data are expressed as mean ± SD. *: $p < 0.05$, **: $p < 0.01$, compared with control. (a, b, c, d, e and f are the quantified data corresponding to Figure 2c, 3c, 4b, 5b, 6 and S3b, respectively.)

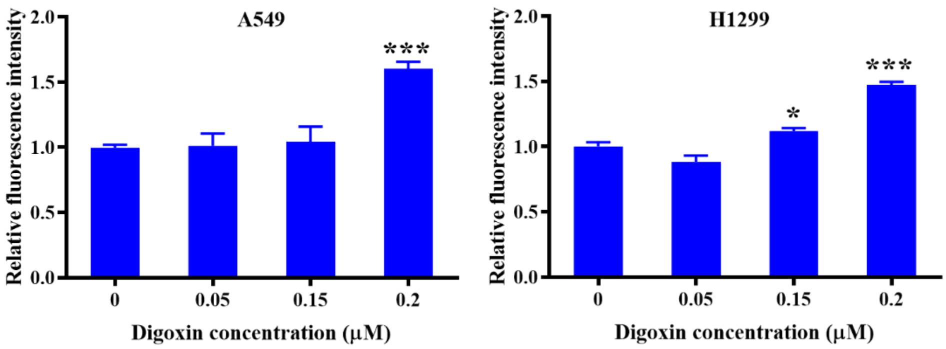

Fig.S6**. Quantification of the fluorescence intensity in A549 and H1299 cells.** The data are expressed as mean ± SD (*n* = 3). *: $p < 0.05$, ***: $p < 0.001$, compared with control. (Corresponding to Figure 5a).

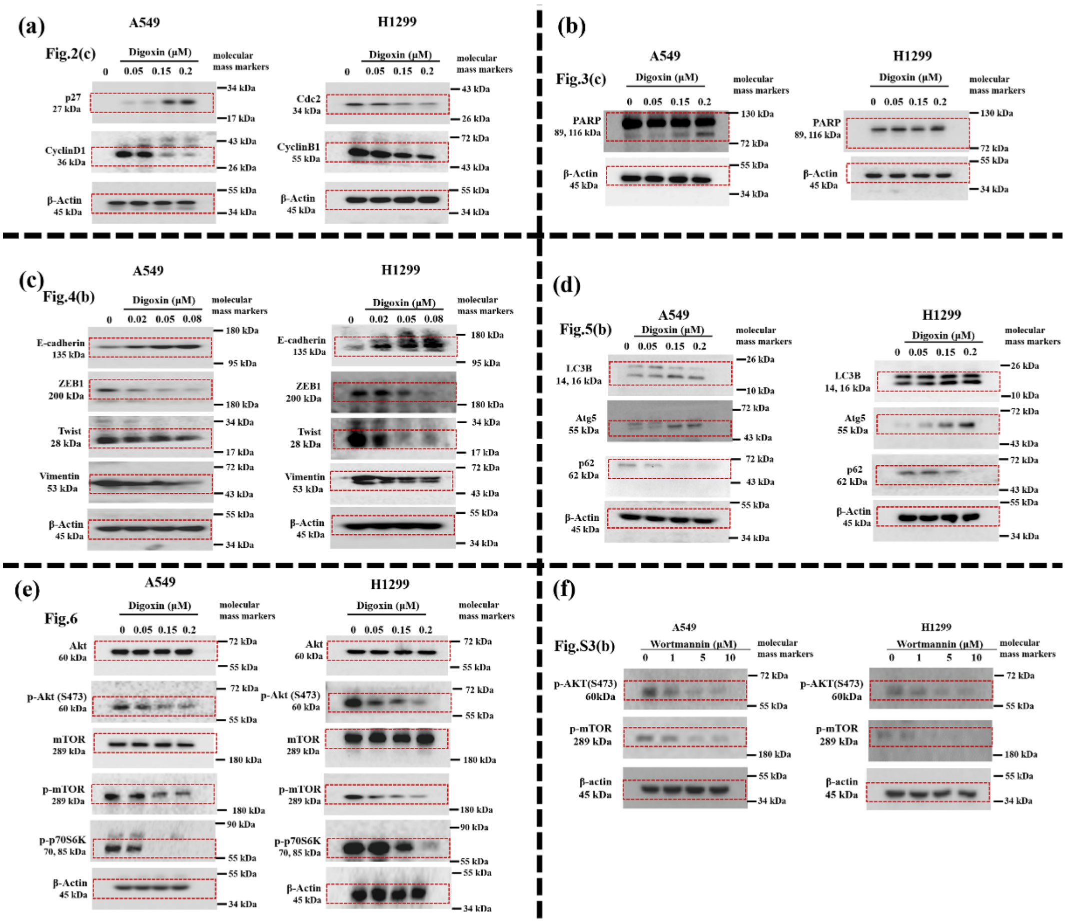

Fig.S7. **The full uncropped raw data of the Western blots including the molecular mass markers.** a, b, c, d, e and f are the raw data corresponding to Figure 2c, 3c, 4b, 5b, d and S3b, respectively.
